# Evaluation of Skin and Soft Tissue Infection Outcomes and Admission Decisions in Emergency Department Patients

**DOI:** 10.1155/2018/7142825

**Published:** 2018-06-13

**Authors:** Nicholas Black, Jon W. Schrock

**Affiliations:** ^1^UCSF Fresno Medical Education Program, Emergency Medicine, Department of Emergency Medicine, 155 N. Fresno, Fresno, CA 93701-2302, USA; ^2^MetroHealth Medical Center, The Department of Emergency Medicine, Case Western Reserve University School of Medicine, 2500 MetroHealth Drive, Cleveland, OH 44109, USA

## Abstract

**Background:**

Skin and soft tissue infections are common presenting complaints for Emergency Department (ED) patients. Although they are common, there remain no definitive guidelines on decisions of admission for these patients.

**Objectives:**

To determine the influence of demographic and clinical information of those presenting with skin and soft tissue infection(s) (SSTI) on both disposition and treatment failure.

**Methods:**

We prospectively enrolled adults with SSTI seen at a large urban ED. Secondary outcome was treatment failure. Statistics utilized* t*-tests and multivariate logistic regression.

**Results:**

We enrolled 125 subjects and 32 were admitted. 15.2% of patients failed treatment with both increasing age and infection area correlating with admission. IV drug use (IVDU) (OR: 10.2; 95% confidence interval [CI]: 1.9 to 50.0) and recent antibiotic use (OR: 2.9; 95% CI 1.003 to 8.333) independently predicted admission. Age and recent surgery in the area of infection (OR: 6.4; 95% CI 1.3 to 30.8) showed positive association with treatment failure. IV antibiotics (OR: 22.3; 95% CI 2.8 to 179.4) and admission (OR: 12.1; 95% CI 2.9 to 50.4) strongly predicted treatment failure.

**Conclusions:**

Age, infection size, IVDU, and recent antibiotics predicted admission. Age, recent surgery at infection site, IV antibiotics, and admission correlated with treatment failure.

## 1. Introduction

The incidence of skin and soft tissue infections (SSTI) in the general population has been increasing in recent years, resulting in a larger number of patients being seen and treated in the Emergency Department (ED), as well as admitted to the hospital [1–3]. Annually, more than four million patients are seen in the ED for SSTI, increased by nearly one million from 2007 [4]. To a large degree, this trend appears to correlate with the greater prevalence of community-associated methicillin-resistant Staphylococcus aureus (CA-MRSA) [5, 6].

Despite the increase in ED SSTI presentations, treatment recommendations remain predominantly nonspecific concerning antibiotic choice and need for admission. The most recent Infectious Diseases Society of America (IDSA) guidelines from 2014 sought to classify patients into mild, moderate, or severe infections, but allow for provider interpretation of systemic signs of illness [7]. However, the only disposition recommendations involved immunocompromise, failure of current treatment, necrotizing infection, and expected unreliable patient adherence. Several studies have previously examined patient and infection characteristics associated with inpatient admission, and one included a focus on provider decision-making with regard to disposition [8–11]. However, there was no clear consensus between the studies. Similarly, there have been attempts to determine characteristics that make treatment failure more likely, but they have not shown any clear associations [10, 12–15].

Given the morbidity, mortality, and cost associated with the increasing SSTI prevalence in the ED, particularly resulting from initial treatment failure, it is clearly important to delineate features associated with more serious disease [16, 17]. This study attempted to determine the influence of provider decision-making and patient presentation on disposition and treatment failure.

## 2. Methods

The study was performed prospectively at a large, urban, county hospital ED with an annual census of more than 100,000 visits. The ED is staffed by practicing emergency physicians, emergency medicine residents, and advanced practice providers 24 hours daily, seven days per week. The study was reviewed and approved by our institutions' Internal Review Board.

Patients were prospectively enrolled from a convenience sample of adults aged 18 or older presenting to the ED with a chief complaint of skin and soft tissue infection (SSTI) including cellulitis, abscess, abscess with cellulitis, carbuncle, and furuncles, over a nine-month period including 2016-17, between the hours of 0800 and 1700. Participant identification and confirmation of eligibility were evaluated through the review of electronic medical records (EMR). Inclusion criteria were defined as age 18 or older, SSTI as the chief complaint, and access to a telephone. Exclusion criteria included diagnosis of gangrene, diagnosis of necrotizing fasciitis, diagnosis of diabetic foot ulcer(s), diagnosis of osteomyelitis, diagnosis of septic arthritis, diagnosis of decubitus ulcer(s), current pregnancy, patient status as a prisoner, and presentation with chief complaint other than SSTI. These diagnoses were excluded if present at the time of ED presentation only. Patients were presented with the details of the study and enrolled after obtaining informed consent.

Vital signs were collected in triage upon admission to the ED. The following data were collected from each patient: demographics; assessment of obesity (defined as BMI ≥ 30); smoking status; alcohol consumption; current IV drug use (IVDU); history of diabetes mellitus, connective tissue disease, liver disease, cancer, renal disease, hidradenitis, chronic edema, venous insufficiency, and chronic leg ulcers/wounds; human bite exposure; mammal bite exposure; antibiotic exposure in the past month for any reason; history of SSTI in the past year; abscess incision and drainage in the past year; and history of surgery in the area of infection.

The maximal area of erythema of the SSTI was measured and assigned to one of the following bodily areas: head, torso, arm, hand, leg, foot, or multiple site. All measurements were performed by a single investigator (NB). If performed, incision and drainage of the SSTI were recorded. Relevant laboratory data obtained throughout the visit were also collected via EMR, including WBC count, ESR, and CRP. Antibiotics administered during the visit, prescribed at discharge, and/or administered during admission were recorded and differentiated by route of administration and specific antibiotic.

The treating physician or provider was then questioned for their patient's final diagnosis: cellulitis, abscess, or abscess with surrounding cellulitis. Each physician or provider was then presented with a survey asking them to identify their intended disposition of the patient (discharge home, admission to the observation unit, admission to the floor, admission to medical step-down, or admission to the intensive care unit) and identify which of the following factors influenced their disposition decision: SSTI area, SSTI location, presence of comorbidities, absence of comorbidities, systemic illness, absence of systemic illness, immunosuppression, or failure of prior treatment. Respondents were then free to provide any additional rationale. If the patient was not discharged home, the provider was asked to provide an anticipated length of stay (LOS).

Each patient was contacted by phone two weeks following their discharge from the index visit to the ED. Patients were contacted three different days by phone for follow-up questions. If we were not able to reach them by phone then medical records were evaluated to determine if a return visit took place. Return visit(s) to an ED for the same infection, changes in discharge antibiotics, and readmission if originally admitted were recorded. Review of the EMR was conducted for those patients admitted to any area of the hospital to document LOS, any changes in antibiotics, and discharge destination: home, skilled nursing facility (SNF), rehabilitation/long-term acute care (LTAC), hospice care, or morgue.

The primary outcomes of the study were the initial disposition of the patient following ED presentation and treatment failure, defined as repeat ED visit for the same SSTI, an antibiotic change within the 2-week follow-up period, readmission to the hospital, or any combination of these factors. Secondary outcomes included LOS, provider reasoning, and anticipated LOS.

All data were entered into a database for analysis and analyzed using Stata 13.0 (StataCorp, LLC, College Station, TX, USA) and MiniTab Express 1.5 (MiniTab Inc., State College, PA, USA). Data are reported as frequencies and medians with interquartile ranges (IQR). Comparisons of groups were performed using the* t*-test and *χ*^2^ test where appropriate.

Demographic and patient characteristics thought to be clinically relevant to disposition decision on descriptive evaluation of the database were evaluated by simple binary logistic regression for assessment of univariate significance. Insignificant characteristics (p < 0.25) were excluded and the following were entered into a logistic regression controlled for age and gender: IVDU, diabetes mellitus, chronic edema, venous insufficiency, chronic leg ulcers, antibiotic use within the past month, and recent surgery at the site of infection. The effects of location of infection on disposition and diagnosis on disposition were also explored via independent logistic regressions, both controlled for age and gender.

Demographic and patient characteristics thought to be relevant to treatment failure (p > 0.25) also underwent univariate analysis, with the following significant variables being placed into a logistic regression controlled for age and gender: antibiotic within the past month, recent surgery in the area of infection, and chronic leg ulcers. Individual logistic regressions were also performed to determine the effect of infection site, diagnosis, antibiotic administration route, and disposition on treatment failure which were reported as odds ratios (OR). All were controlled for age and area of infection.

## 3. Results

125 patients were initially enrolled in the study and there were no subsequent exclusions. Median age was 39 years (interquartile range [IQR]: 31-56 years). Males comprised 69 (55.2%) of patients. Ten participants (8.0%) were identified as IV drug users. The most commonly reported comorbidity was chronic edema in 30 patients (24.0%). Also reported were 16 (12.8%) patients with diabetes mellitus, 10 (8.0%) with liver disease, 14 (11.2%) with venous insufficiency, and 7 (5.6%) with chronic leg ulcers. Forty patients (32.0%) had received antibiotics within the month prior to presentation, 50 (40.0 %) reported a SSTI within a year prior to presentation, and 18 (14.4%) had recent surgery in infection. Other demographic data and participant characteristics are shown in Table 1. Forty-eight patients (38.4%) were diagnosed with cellulitis, 47 (37.6%) with an abscess, and 30 (24.0%) with an abscess complicated by surrounding cellulitis. The median area of infection in all participants was 35 cm^2^ (IQR: 9.0-124.0 cm^2^). The head was the location of SSTI in nine patients (7.2%), torso in 20 (16.0%), arm in 22 (17.6%), hand in 10 (8.0%), leg in 35 (28.0%), foot in 11 (8.8%), and multiple sites in 18 (14.4%). Further clinical characteristics of participants are found in Table 2. Antibiotics received in the ED can be seen in Table 3.

Of the 125 patients enrolled, 32 (25.6%) were admitted: 8 (25.0%) to the CDU and 24 (75.0%) to the medical floor. There were no step-down or medical ICU admissions. Among those admitted, median age was 58 years (IQR: 37-68 years, mean: 54.9 years) and 18 (56.3%) were male. Selected comorbidities of admitted participants were diabetes mellitus in 9 (28.1%), liver disease in 4 (12.5%), chronic edema in 15 (46.9%), venous insufficiency in 9 (28.1%), and chronic leg ulcers in 5 (15.6%). Sixteen (50.0%) had received antibiotics in the prior month and 15 (46.9%) had been diagnosed with a SSTI in the prior year. Nine (28.1%) had undergone recent surgery in the SSTI. Further demographics and background statistics of those admitted are also found in Table 1. Diagnosis in admitted patients was cellulitis in 20 (62.5%), abscess in 4 (12.5%), and abscess with complicating cellulitis in 8 (25.0%). Median area of infection was 319.0 cm^2^ (IQR: 67.0-891.0 cm^2^, mean: 725.6 cm^2^) and the leg was the most frequent site (n=11, 34.4%). Median LOS was 2 days (IQR: 1-3.5 days, range: 1-15 days).

For the 32 patients that providers judged as requiring admission, providers noted that infection size influenced their decision in 14 (43.8%) cases, infection location in 6 (18.9%) cases, presence of comorbidities in 18 (56.3%), systemic signs of infection in 7 (21.9%) cases, immunosuppression in 2 (6.3%) cases, and failure of prior treatment in 9 (28.1%) cases. Physician reasons for admission can be seen in Table 4. Median anticipated LOS was 2 days (IQR: 1-3 days, range: 1-4 days).

Follow-up contact was made with 77 (61.6%) of the participants; for those who failed telephone follow-up, the EMR was reviewed and no return ED visits were found for the same infection. Treatment failure was demonstrated in only 19 (24.7%) of those contacted. Of those reporting treatment failure, 16 (84.2%) had a change in antibiotics, 4 (21.2%) returned to the ED, and 3 (15.8%) required repeat hospitalization. Median age of patients failing treatment was 58 years (IQR: 36-68 years, mean: 55.0 years) while the median age of those with successful clinical treatment was 39 years (IQR: 30-50 years, mean: 41.7 years). Median area of infection was 142.0 cm^2^ (IQR: 35.0 -700.0 cm^2^, mean: 625.7 cm^2^) for those failing treatment and 24.3 cm^2^ (IQR: 7.0-67.0 cm^2^, mean: 124.0 cm^2^) in patients successfully treated.

Ninety-three (74.4%) participants received a discharge home. The median age was 39 years (IQR: 29-49 years, mean: 43.3 years) for those discharged home. Two-sample* t*-test comparison of the mean ages of admitted patients and home discharges demonstrated a significantly increased age among those admitted (p=0.0002). Area of infection in the home discharge group showed a median of 19.6 cm^2^ (IQR: 0.03-50.24 cm^2^, mean: 87.57 cm^2^). Two-sample* t*-test comparison of infection area between this group and admitted patients also confirmed a significantly increased area of infection in those admitted (p=0.0059). IV drug users (OR: 10.2, 95% CI: 1.9-50.0) and patients who had taken antibiotics in the month prior to presentation (OR: 2.9, 95% CI: 1.003-8.333) were significantly more likely to be admitted when controlled for age, gender, recent surgery around infection, and comorbidities of diabetes mellitus, chronic edema, venous insufficiency, and chronic leg ulcers. In this model, increasing age was also demonstrated to have a positive association with admission (OR: 1.06, 95% CI: 1.02-1.09). Neither site of infection nor diagnosis was significantly related to disposition after controlling for age and area of infection, though area of infection was associated with an increased risk of admission in the model (OR: 1.002; 95% CI: 1.001-1.004). A one-sample* t*-test of the difference between anticipated and actual LOS (mean: 0.6 days) performed against an expected difference of zero was significant, p=0.04.

Two-sample* t*-test comparison revealed a significantly higher mean age of those failing treatment than receiving successful treatment (p=0.0105) but failed to reveal a significant difference in the area of infection (p=0.0828). Patients with recent surgery around infection were more likely to fail treatment (OR: 6.4, 95% CI: 1.3-30.8) when controlled for age, gender, antibiotics within the prior month, and chronic leg ulcers. Within this model, increasing age was shown to be a factor favoring admission (OR: 1.05, 95% CI: 1.01-1.08). Logistic regression failed to show an increased likelihood of treatment failure based on site of infection or diagnosis when controlled for age and area of infection. The area of infection also did not demonstrate an associated risk of treatment failure. However, treatment failure was more likely in those receiving IV antibiotics in the ED (OR: 22.3, 95% CI: 2.8-179.4) and those admitted (OR: 12.1, 95% CI: 2.9-50.4) when controlled for age and size of infection. For our cohort, the number of patients admitting IV drug use was relatively small.

## 4. Discussion

Our data demonstrated an overall admission rate of 25.6% between the CDU (6.4%) and medical floor (19.2%). This rate of admission was greater than prior studies hoping to elucidate factors associated with patient disposition [8, 11]. However, a prior study at the same institution did note a slightly higher rate of admission to the CDU and medical floor, 42.8% [10]. It is reasonable to assume that the patient population of the enrolling institution may harbor greater illness severity given its position as a major safety-net provider for the area and that illness severity in this cohort was reduced as compared to prior due to convenience sampling.

Our data showed that the age of the patient population admitted to either the CDU or the medical floor was significantly greater than that of the population discharged to home. Age has been both replicated and discounted as a factor in admission [8–11]. Along with age, IVDU and exposure to antibiotics in the past month were associated with admission. IVDU has been investigated in prior papers and found not to be a factor [10]. The findings of this study may reflect the newest IDSA guidelines suggesting admission in the face of expected patient nonadherence often demonstrated by those suffering addiction.

Recent antibiotic use has also been demonstrated in multiple cohorts to be a predictor of inpatient admission [12, 14]. Admission following outpatient treatment failure has been found to be quite common and providers in this study frequently cited failure of prior treatment as a factor in their decision to admit [8]. The demonstrated relationship between increasing size of infection and decision to admit is not novel and was also frequently reported by study providers to have been a factor in their decision [8].

It was surprising that no particular comorbidity or location of infection was associated with the decision not to discharge a patient to home. However, Talan et al. found a significant relationship between the presence of any comorbidity and admission [8]. It is likely that had these been examined in aggregate in our study, the result would be similar. Provider decision-making frequently referenced the presence of a comorbidity. Volz et al. cited infection location, particularly the hand, as an independent predictor of admission from the CDU by a large odds ratio, though this differs from our data that did not demonstrate any association [11]. We would have expected the prevailing dogma regarding conservative management of SSTI of the hand to be reinforced; however, in our cohort, provider decision to admit was infrequently supported by location. This discrepancy remains unclear.

LOS was significantly longer than the anticipated length of stay in our study, though by less than one day. This could be a result of the admitted patients suffering much more severe disease than is typical, provider underestimation of illness severity, or minor systems-based issues in coordination of patient care. The true cause is not clear from the data.

Our treatment failure rate of 24.7% is comparable with that found in other studies that examined outpatient, inpatient, and ED treatment failure, which ranged from 18.7% to 32.1% [12–15, 18]. From this comparison, it can be assumed that the treatment success rate and practice patterns of the providers in the study are likely valid.

The measured size of the area of infection was not significantly associated with likelihood of treatment failure in our study. Prior studies have reached mixed conclusions, but it appears that physician use of infection size as a decision point for admission may be warranted given this result [13, 14]. Location of infection also did not demonstrate significance regarding failure of ED treatment. It was also unexpected that univariate and multivariate analysis did not reveal specific comorbidities associated with treatment failure, as other studies have shown correlations with obesity, chronic lower extremity edema, and chronic lower extremity ulceration [12, 15]. We also would have expected to see an effect on treatment failure likelihood with recent antibiotic use based on previous studies and the increased odds of admission detected in our study [12, 13]. This may reflect changing practice patterns and improved antibiotic selection in the face of continuing pressure from resistant organisms, most notably CA-MRSA.

Perhaps most interestingly, the odds of treatment failure were significantly increased with both IV antibiotics and admission. Along with the study data suggesting longer than anticipated stays in the hospital, this result could point even more strongly toward provider underestimation of illness severity in patients they deem worthy of admission. Another explanation is that the choice of antibiotic was left to the discretion of the treating provider, with significant variability between providers known to exist. This is likely to be especially true between the ED and medical floors where a switch from broad spectrum to narrower spectrum antibiotics is often made. Supporting this reasoning is the fact that most of treatment failures in this study resulted from antibiotic changes. Interplay between the initial infective species and the influence of resistant organisms in the hospital environment can also not be discounted. Given the ambiguity in interpreting this information, further study is warranted on this matter.

This study does have some limitations which should be noted. Despite the strength of the prospective study design, the single enrollment center, small number of participants, and the necessity of enrolling patients by convenience sample within an established hourly time frame allow significant possibility of sampling bias. The low successful follow-up rate may also lead to a sampling bias, most directly affecting analysis of treatment failure. The majority of those lost to follow-up were discharged home and presumably less likely to fail treatment, resulting in a greater proportion of inpatient treatment failure to outpatient treatment failures than might otherwise be expected. Finally, the study cohort was predominantly discharged home, with no patients admitted to step-down or medical ICU. This indicates that conclusions drawn from the cohort may not be generalizable to an ED population with a higher severity of illness. Our rates of patients who admit to IV drug use may be different than other communities.

## 5. Conclusions

Our study found that increasing age, size of infection, IVDU, and recent antibiotic use were correlated with an increased likelihood of admission to the CDU or medical floor, while previously demonstrated predictors such as individual comorbidities and infection location did not. Despite this lack of association, providers often cited patient comorbidity as a factor in their decision. Size of infection was also heavily cited as a factor in the decision to admit. Treatment failure was once again positively correlated with age but did not demonstrate association with size of infection or prior antibiotic use. Our data were differentiated from prior work in that recent surgery around infection, IV antibiotic administration, and admission were independently associated with treatment failure to a great degree. The reason for these associations is not clear and would require further investigation.

## Figures and Tables

**Table 1:** Demographic data and characteristics of enrolled patients.

**Characteristics**	**All Patients (n=125)**	**Admitted (n=32)** ^**a**^
Age (years; median [IQR])	39.0 [31.0, 56.0]	58.0 [36.5, 67.8]
Gender - n (%)		
Male	69 (55.2)	18 (56.3)
Obese - n (%)	39 (31.2)	7 (21.9)
Smoking - n (%)	72 (57.6)	17 (53.1)
>15 Alcoholic Drinks per Week - n (%)	9 (7.2)	1 (3.1)
IV Drug Use - n (%)	10 (8.0)	5 (15.6)
Comorbidities - n (%)		
Diabetes Mellitus	16 (12.8)	9 (28.1)
Liver Disease	10 (8.0)	4 (12.5)
Active Cancer	2 (1.6)	1 (3.1)
Renal Disease	3 (2.4)	2 (6.3)
Hidradenitis	5 (4.0)	1 (3.1)
Chronic Edema	30 (24.0)	15 (46.9)
Venous Insufficiency	14 (11.2)	9 (28.1)
Chronic Leg Ulcers/Wounds	7 (5.6)	5 (15.6)
Human Bite - n (%)	0 (0.0)	0 (0.0)
Mammal Bite - n (%)	6 ( 4.8)	2 (6.3)
Antibiotics in the Past 4 Weeks - n (%)	40 (32.0)	16 (50.0)
SSTI in the Past Year - n (%)	50 (40.0)	15 (46.9)
Abscess Drainage in the Past Year - n (%)	27 (21.6)	6 (18.8)
Prior Surgery in Area of SSTI - n (%)	18 (14.4)	9 (28.1)

^a^Admitted to CDU or medical floor.

**Table 2:** Clinical characteristics of enrolled patients.

**Characteristics**	**All Patients (n=125)**	**Admitted (n=32)** ^**a**^
Vitals (median [IQR])		
Temperature (C)	36.8 [36.6, 36.9]	36.8 [36.6, 37.0]
Systolic Blood Pressure (mmHg)	135.0 [123.0, 145.0]	139.0 [127.3, 152.3]
Diastolic Blood Pressure (mmHg)	79.0 [70.0, 86.0]	79.0 [67.8, 88.5]
Heart Rate (1/min)	83 [75, 92]	81 [71, 97]
Oxygen Saturation (%)	98.0 [97.0, 99.0]	97.5 [96.0, 98.8]
Diagnosis - n (%)		
Cellulitis	48 (38.4)	20 (62.5)
Abscess	47 (37.6)	4 (12.5)
Abscess with Surrounding Cellulitis	30 (24.0)	8 (25.0)
Area of Infection (cm^2^; median [IQR])	35.0 [9.0, 124.0]	319.0 [67.0, 891.0]
Location - n (%)		
Head	9 (7.2)	1 (3.1)
Torso	20 (16.0)	1 (3.1)
Arm	22 (17.6)	4 (12.5)
Hand	10 (8.0)	2 (6.3)
Leg	35 (28.0)	11 (34.4)
Foot	11 (8.8)	5 (15.6)
Multiple Site	18 (14.4)	8 (25.0)

^a^Admitted to CDU or medical floor.

**Table 3:** Antibiotics administered in the ED.

**Antibiotic Classification**	**n (**%**)**^**a**^
Penicillins - n (%)	10 (8.0)
Cephalosporins - n (%)	23 (18.4)
Bactrim - n (%)	36 (28.8)
Clindamycin - n (%)	43 (34.4)
Doxycycline - n (%)	7 (5.6)
Vancomycin - n (%)	20 (16.0)
Other^b^ - n (%)	5 (4.0)
None - n (%)	9 (7.2)

^a^15.2% of patients received multiple antibiotics.

^b^Including fluoroquinolones (n=2), metronidazole (n=1), and mupirocin (n=2).

**Table 4:** Physician reasoning for disposition decisions.

**Reasoning** ^**a**^	**Home D/C (n=93)**	**Admitted (n=32)** ^**b**^
Infection Size - n (%)	66 (71.0)	14 (43.8)
Infection Location - n (%)	23 (24.7)	6 (18.8)
Comorbidities - n (%)	0 (0.0)	18 (56.3)
Absence of Comorbidities - n (%)	55 (59.1)	0 (0.0)
Systemic Signs - n (%)	0 (0.0)	7 (21.9)
Absence of Systemic Signs - n (%)	2 (2.2)	0 (0.0)
Immunosuppressed Patient - n (%)	0 (0.0)	2 (6.3)
Failure of Prior Treatment - n (%)	0 (0.0)	9 (28.1)
Multiple reasons - n (%)	81 (87.1%)	24 (75.0%)

^a^More than one reason could be chosen for each patient.

^b^Admitted to CDU or medical floor.

## Data Availability

Datasets will not be made available.
